# Impact of temperature shifts on the joint evolution of seed dormancy and size

**DOI:** 10.1002/ece3.2611

**Published:** 2016-11-27

**Authors:** Yang Liu, Sébastien Barot, Yousry A. El‐Kassaby, Nicolas Loeuille

**Affiliations:** ^1^ Department of Forest and Conservation SciencesUniversity of British ColumbiaVancouverBCCanada; ^2^ Sorbonne UniversitésInstitute of Ecology and Environmental Sciences (UMR 7618, UPMC, CNRS, INRA, IRD)ParisFrance

**Keywords:** climate change, eco‐evolutionary dynamics, life‐history traits, seed dormancy, seed size, structured population model, temperature shifts and fluctuations

## Abstract

Seed dormancy and size are two important life‐history traits that interplay as adaptation to varying environmental settings. As evolution of both traits involves correlated selective pressures, it is of interest to comparatively investigate the evolution of the two traits jointly as well as independently. We explore evolutionary trajectories of seed dormancy and size using adaptive dynamics in scenarios of deterministic or stochastic temperature variations. Ecological dynamics usually result in unbalanced population structures, and temperature shifts or fluctuations of high magnitude give rise to more balanced ecological structures. When only seed dormancy evolves, it is counter‐selected and temperature shifts hasten this evolution. Evolution of seed size results in the fixation of a given strategy and evolved seed size decreases when seed dormancy is lowered. When coevolution is allowed, evolutionary variations are reduced while the speed of evolution becomes faster given temperature shifts. Such coevolution scenarios systematically result in reduced seed dormancy and size and similar unbalanced population structures. We discuss how this may be linked to the system stability. Dormancy is counter‐selected because population dynamics lead to stable equilibrium, while small seeds are selected as the outcome of size‐number trade‐offs. Our results suggest that unlike random temperature variation between generations, temperature shifts with high magnitude can considerably alter population structures and accelerate life‐history evolution. This study increases our understanding of plant evolution and persistence in the context of climate changes.

## 1 Introduction

Selection in variable environments may favor plants to synchronize seed dispersal with environmental conditions allowing germination or defer germination until suitable conditions occur (Freas & Kemp, 1983). Seed dormancy is an innate constraint on germination timing under conditions that would otherwise promote germination in nondormant seeds (Simpson, 1990) and prevents germination during periods that are ephemerally favorable (Bewley, 1997). Timing of seed germination is the earliest trait in plant life history, allowing plants to regulate when and where they grow. It affects the evolution of other life‐history traits that follow in the life cycle, such as fecundity and survival (Hamilton, 1966). As such, seed dormancy may be construed as an adaptation for survival during bad seasons and can exert cascading selective pressures on subsequent life stages.

Plants bear seeds with a spectrum of dormancy intensities (Baskin & Baskin, 1998) and distribute their offspring across time, hedging their bets against unpredictable environments (Poisot, Bever, Nemri, Thrall, & Hochberg, 2011; Venable, 2007). This increases the likelihood that some seeds will survive regardless of environmental variations. Seed dormancy variability among individuals is associated with environmental heterogeneity (Angevine & Chabot, 1979) and heterogeneous environments may select for bet‐hedging strategies, as population growth is an inherently multiplicative process that is very sensitive to occasional extreme values (Dempster, 1955). Cohen (1966) indicated that low germination probabilities can be expected in harsh environments as individuals can germinate in improved conditions and decrease their average mortality (Cohen, 1966). However, Ellner (1985a, 1985b) predicted that increasing the frequency of favorable years may also lead to lower germination rates due to increased density‐dependent effects imposed by competitive interactions (Ellner, 1985a, 1985b).

In contrast to periodic fluctuations of good and bad seasons among years, climate change increases the probability of bad seasons for initially locally adapted phenotypes, as environments continuously move away from past optimums. Predictably, air temperatures will increase by 0.8–1.0°C in 2050s and by 2–4°C in 2100s (IPCC 2007). Such a warming is expected to reduce seedling emergence (Cochrane, Holye, Yates, Wood, & Nicotra, 2015; Hoyle et al., 2013). On the other hand, the evolution of seed dormancy is favored by high seed persistence in the soil seed bank to alleviate the cost of delayed germination (Childs, Metcalf, & Rees, 2010). Both Cohen and Ellner's models suggested that an increase in seed survivorship selects a low seed germination (Cohen, 1966; Ellner, 1985a, 1985b). Climate change engenders long‐term exposure to high soil temperatures, which may reduce seed survival, thus selecting for lower levels of seed dormancy (Ooi, Auld, & Denham, 2009). Taken together, climate change may increase seed numbers in life cycle and decrease dormancy levels due to increased seed mortality.

Seed size is another crucial life‐history trait that links the ecology of reproduction and seedling establishment with that of vegetative growth. Seed size commonly varies over five to six orders of magnitude among coexisting plant species (Leishman, Wright, Moles, & Westoby, 2000). Seed size is closely correlated with changes in plant form and vegetative type, followed by dispersal syndrome and net primary productivity (Moles et al., 2005, 2007). Effects of temperature on seed size are not consistent, as both increased (Liu, Wang, & El‐Kassaby, 2016; Murray, Brown, Dickman, & Crowther, 2004) or reduced (Hovenden et al., 2008) seed sizes have been documented. Production of dimorphic or heteromorphic seeds by a single plant allows plants to decrease temporal variance in offspring success through bet‐hedging (Venable, Búrquez, Corral, Morales, & Espinosa, 1987). The diversity of seed size may be maintained by tolerance–fecundity trade‐offs (i.e., more tolerant (fecund) species gain more (less) stressful regeneration sites, respectively) (Muller‐Landau, 2010). The role of differential seed size in promoting species coexistence has been stressed by previous theoretical studies (Geritz, 1995; Geritz, van der Meijden, & Metz, 1999; Rees & Westoby, 1997). Large seed size confers direct advantages to many fitness‐related plant characteristics, including recruitment and survivorship (Mcginley, Temme, & Geber, 1987; Moles & Westoby, 2004), and establishment (Leishman et al., 2000; Moles & Westoby, 2004) because large seeds accumulate copious nourishing substances for germination and have better tolerance in face of disturbances (e.g., abiotic stresses) (Geritz et al., 1999; Westoby, Falster, Moles, Vesk, & Wright, 2002). On the other hand, for a given reproductive investment, seed size is negatively correlated with seed number (Harper, Lovell, & Moore, 1970; Jakobsson & Eriksson, 2000; McGinley & Charnov, 1988) and large seeds are less dispersible due to their great size (Salisbury, 1975).

Although the evolution of seed dormancy and size was modelled separately, variation in seed size (morphology) often has a concomitant effect on seed dormancy (reviewed by (Baskin & Baskin, 1998)). Lines of genetic evidence underpin that during development, physiological seed dormancy and seed size are regulated by phytohormone signaling pathways, which have opposite effects on seed dormancy and size (Footitt, Douterelo‐Soler, Clay, & Finch‐Savage, 2011; Hu et al., 2008), thus suggesting that they evolve in a coordinated manner. Also, some common selective pressures are likely to affect seed dormancy and size simultaneously, such as light, water availability or potential, and intraspecific competition (Baskin & Baskin, 1998; Larios, Búrquez, Becerra, & Venable, 2014). Owing to environmental pressures (e.g., frost, drought), species that produce light seeds are more likely to possess some type of seed dormancy (morphological, physiological, physical, morphophysiological, or physiophysical) (Rees, 1993; Venable & Brown, 1988) and a negative relationship between seed dormancy and size was documented in many cases, although this pattern is not universal (Grime et al., 1981; Kiviniemi, 2001; Larios et al., 2014; Rees, 1996; Thompson & Grime, 1979; Vidigal et al., 2016). These inconsistencies may be explained by an incomplete consideration of other co‐varying factors (e.g., dispersal, fire, predation) (Rees, 1996) or by phylogenetic constraints (Willis et al., 2014). Additionally, germination of large‐seeded species is strongly facilitated by temperature fluctuations, ensuring germination after deep burial or in litter layers (Ghersa, Arnold, & Martinezghersa, 1992; Pearson, Burslem, Mullins, & Dalling, 2002; Xia, Ando, & Seiwa, 2016).

In this article, we model and parameterize a stage‐structured population to study the impact of changing temperatures on the joint and independent evolution of seed dormancy and size. Altering temperature leads to an enlarged mismatch of a species’ eco‐evolutionary trajectory in its actual living habitat and the environment to which it is best suited. We incorporate the impact of temperature on germination success. Furthermore, analyses of evolutionary speed of the two traits enable us to see whether evolutionary responses are sufficient to offset negative effects of shifting climate (i.e., revolutionary rescue (Gonzalez, Ronce, Ferrière, & Hochberg, 2013)). Under evolutionary forces driven by interplays between environments and life‐history traits, we aim to investigate:

1. The effects of temperature shifts on the evolution of seed dormancy and size. Global change, by producing increasingly frequent bad years, should select for dormancy. However, when germination success is negatively affected, the number of seeds may increase in the soil seed bank, thus increasing mortality through density‐dependent effects. We here investigate which of the two antagonistic mechanisms dominate in the evolution of seed dormancy. Moreover, we expect that temperature shifts will be less conducive to the evolution of seed size as fecundity benefits of reduced seed size can offset survival costs in the context of environmental change, so that temperature shift does not change the overall balance of benefits and costs.
2. Whether evolutionary dynamics differ when we allow for a joint evolution of the two traits (scenarios subject to coevolution). As per empirical observations, we expect the joint evolution to yield a negative correlation between the two traits (Grime et al., 1981; Kiviniemi, 2001; Larios et al., 2014; Rees, 1996; Thompson & Grime, 1979; Vidigal et al., 2016).
3. Effects of the evolution on the ecological structure at the population level (relative abundance of seeds and adults). We expect that (1) decreases in seed dormancy will increase the number of adults relative to seeds, because the probability of germination increases (note: constant adult survival assumed) while seed survival decreases; and (2) changes on seed size will not significantly alter the population structure, because seed size affects seed survival and fecundity in opposite ways. Nonetheless, maladaptation caused by temperature shifts or fluctuations interacts with evolution and may have a great impact on the population structure. We expect that in temperature shifts, the number of seeds relative to adults will increase, thus leading to more balanced population structure, while the total population density will shrink due to the altered environment. The probability of germination greatly decreases particularly at wide temperature shifts, resulting in less adults, while elevated fecundity due to relaxed adult density‐dependent competition (and predictably smaller seeds, if seed size evolves or coevolves with seed dormancy) results in more seeds.

## 2 The model

### 2.1 Description of the ecological model

**Figure 1:**
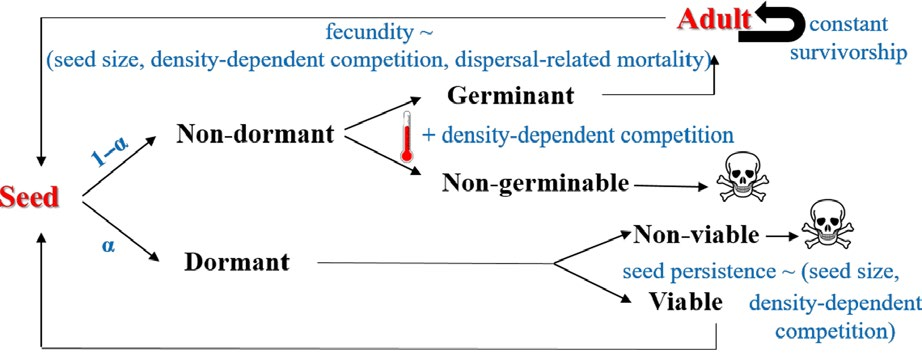
Life cycle of seed‐adult stages. Note: α denotes the probability of dormant seeds

We model the dynamics of a two‐stage population (seeds and adults) under the assumption that density‐dependent competition affects seed survival, germination, and adult fecundity, using Ricker functions (Ricker, 1954). We assume that temperature constrains germination, as seedling is the most fragile phase and the temperature for seed emergence is important in plant life histories. The local dynamics in seed, *S*, and adult, *A*, populations for a given morph *j* are described by the following recursion equations in matrix form:

(1) $$\left(\begin{array}{c} S_{j}\left[t+1\right]\\ A_{j}\left[t+1\right] \end{array}\right)=T_{j}\left(\begin{array}{c} S_{j}\left[t\right]\\ A_{j}\left[t\right] \end{array}\right)=\left(\begin{array}{cc} V_{Sj} & Y_{j}\\ G_{j} & V_{Aj} \end{array}\right)\left(\begin{array}{c} S_{j}\left[t\right]\\ A_{j}\left[t\right] \end{array}\right)$$

where *T*_*j*_ is transitional matrix; *V*_S*j*_, *Y*_*j*_, *G*_*j*_, and *V*_A*j*_ represent seed survival, fecundity (yield), germination, and adult survival, respectively. We assume that seed dormancy α affects seed survival *V*_S*j*_ and germination *G*_*j*_, while seed size γ affects seed survival *V*_S*j*_ and fecundity *Y*_*j*_. Figure 1 delineates the life cycle of seed adult, and Table 1 summarizes the model's variables and parameters. Note that seed size is equivalent to seed mass thereafter.

**Table 1:** Variable/parameter symbols and values used in simulations

Symbol	Variables/parameters	Value/range	Note/unit
α	Seed dormancy	[0.01, 0.99]	Probability, (0, 1);α_r_: resident α, α_m_: mutant α, α: either resident or mutant α
γ	Seed size (mass)^a^	[0.01, ∞)	Weight unit;γ_r_: resident γ, γ_m_: mutant γ, γ: either resident or mutant γ

*abc*	Intensity of density‐dependent competition	0.0010.0020.003	Per individual
*B*	Dispersal‐related probability of mortality when seed size is one	3/5; (0, 1)	Dimensionless
*d*_β_(γ)	Dispersal (β)‐related mortality probability	(0, 1)	Dimensionless
*N*	The total number of morph types	[1, ∞)	In numbers; morph types range from 1st to *k*th
*pquv*	Shape parameters for the function describing how germination depends on seed size	0.810.10.4	Arbitrary, *p* and *q* are dimensionless, and the unit of *u* and *v* is (weight unit)‐1; as surviving and dormant seed is a probability, *p*/*q*,* u*/*v* ∈ (0, 1); as seed size positively correlated with seed survival, we assume *p*/*q *> *u*/*v*.
*V*_*A*0_	Adult survival probability	0.93; (0, 1]	The basic value assumes perennial species
*t*	Generation (simulation) time	1.0 × 10^8^	Number of generations
*T*	Patch temperature	*T*_opt_ ‐ 25°C*T*_*x*_—temperature in a local patch (*T*_opt_ ± 1.5 or 3°C)	°C
θ	Seed size‐related survival probability in the soil seed bank	(0.25, 0.80)	Dimensionless
ω	Investment in reproduction	10	Weight unit
η	Niche width	3; (0, ∞)	°C

a We define that large seeds are those that can contribute to higher than 70% of seed survival rate in the soil seed bank.

(2) $$V_{Sj}=\alpha_{j}\times \theta \times e^{-\sum_{k=1}^{N}\alpha_{jk}S_{k}}=\alpha_{j}\times \frac{p\gamma_{j}+u}{q\gamma_{j}+v}\times e^{-\sum_{k=1}^{N}\alpha_{jk}S_{k}}$$

For a given morph *i*, from Equation (2), α_*j*_ is the basic probability of surviving while dormant in the soil seed bank from a time step to another. This basic mortality is modulated by the effects of seed size, which is incorporated in the second term of the equation. The function we use is monotonically increasing with seed size, given the parameter constraints listed for *p, q*,* u*, and *v* in Table 1. Hence, we assume that larger seeds survive better (Geritz et al., 1999; Westoby et al., 2002). Finally, the probability of survival is reduced by seed density‐dependent effects (e.g., due to resource competition, seed predator attraction and/or foraging (Janzen, 1970, 1971; Charnov, 1976)), which is modelled by the third term of Equation (2). Note that because *V*_S*j*_ is a probability, it is necessary that its maximum α_j_*p/*q* is below one.

(3) $$Y_{j}=\frac{\omega }{\gamma_{j}}\times e^{-\sum_{k=1}^{N}b_{jk}A_{k}}\times \left[1-d_{\beta }\left(\gamma_{j}\right)\right]=\frac{\omega }{\gamma_{j}}\times e^{-\sum_{k=1}^{N}b_{jk}A_{k}}\times \left(1-B^{\gamma_{j}}\right)$$

From Equation (3), fecundity *Y*_*j*_, is constrained by ω, the total reproductive investment of the plant, which is distributed among seeds given seed size γ_*j*_ (Harper et al., 1970; Jakobsson & Eriksson, 2000; McGinley & Charnov, 1988). Fecundity is adult density dependent ((Ellner, 1987), but mathematically differently reflected), as reflected by the second term of the equation. The third term of the equation depicts the probability that seeds are retained locally, which increases with seed size (Salisbury, 1975).

(4) $$G_{j}=\left(1-\alpha_{j}\right)\times e^{-\sum_{k=1}^{N}\left(1-\alpha_{rjk}\right)c_{jk}S_{k}}\times e^{\left[-\frac{1}{2}{\left(\frac{T_{\mathrm{opt}}-T_{x}}{\eta }\right)}^{2}\right]}$$

From Equation (4), (1) − α_j_ is the probability of germination. Success of germination is reduced by juvenile seedling density‐dependent competition, embodied by the second term of the equation. We assume that seed germination hinges on the difference between the optimal germination temperature *T*_opt_ and the actual local temperature in the patch *T*_*x*_. We do not consider the correlation between germination vigor and seed size. Note that the function with respect to the temperature difference is monotonically decreasing so that germination probabilities are reduced when temperature differs more from the optimum. This relationship is modulated by the third term of Equation (4).

(5) $$V_{Aj}=V_{A0}$$

Equation (5) represents the probability of adult survival, *V*_A*j*_. As we are simply interested in how seed traits evolve in response to germination constraints and do not account for how adult survival influences evolution, we assume constant *V*_A*j*_.

### 2.2 Investigations of eco‐evolutionary dynamics

As the stage‐structured model involves complex nonlinear functions of phenotypical traits, analytical investigation is not possible. We therefore rely on extensive simulations and graphical analyses to understand evolutionary dynamics. Overall, three scenarios were considered: (i) evolution of seed dormancy α, (ii) evolution of seed size γ, and (iii) joint evolution of the two traits.

- * Evolution of seed dormancy*

We investigate the adaptive dynamics of seed dormancy using pairwise invasibility plots (hereafter PIPs). These plots display the relative fitness of rare mutants within resident populations, thereby allowing assessments of evolutionary dynamics (Dieckmann & Law, 1996; Geritz, Kisdi, Meszéna, & Metz, 1998) and characterizing evolutionary singularities (i.e., points at which the fitness gradient vanishes (Geritz et al., 1998)). Analyses of PIPs assume that (1) the resident population is at stable equilibrium; (2) reproduction is clonal; and (3) the mutant population is rare. To overcome these restrictive hypotheses (Dieckmann & Law, 1996; Geritz et al., 1998), we undertake extensive numerical simulations to construct evolutionary trajectories of seed dormancy over time.

To build PIPs, we set a spectrum of residents of seed dormancy phenotype whose trait values vary from 0.01 to 0.99 with an interval of 0.01 (i.e., 99 discrete traits). The ecological equilibria of seed, S*, and adult, A*, for those traits are accordingly calculated. We then test the possibility of invasion of each resident phenotype by rare mutants. Possibility of invasion is evaluated by the long‐term growth rate of the population of mutant seeds and adults when rare. The leading eigenvalue (λ_*L*_) of the transitional matrix *T*_*j*_ in Equation (1) is used to approximate the long‐term growth rate. By definition, a successful invader has a λ_*L*_ strictly superior to one. Computations are carried out on *Mathematica* 10.3 (Wolfram Research Inc. 2015).

While PIPs graphically illustrate configurations of evolutionary singularities, they implicitly assume a separation of evolutionary and ecological timescales, as the resident population has to reach the equilibrium before a new mutation occurs. Many empirical observations however suggest that evolution may be as fast as ecological dynamics (Hairston, Ellner, Geber, Yoshida, & Fox, 2005). To relax this limitation, we employ numerical simulations of seed dormancy, in which mutants are introduced with a given probability at each time step, even if the resident population is not at equilibrium. The extent to which ecological and evolutionary timescales overlap may be directly manipulated via altering the probability of mutations. We simulate a span of 1.0 × 10^8^ time steps, and initial resident trait values (i.e., α_r_ and γ_r_) are both 0.5 while initial population size for seeds and adults are both 5. In each time step, phenotypical trait α can randomly mutate. Mutation takes place at a fixed probability (baseline: 10^−8^) and affects a single seed of a resident population. The value for mutants α_m_ is randomly drawn from a uniform distribution centered on the parent trait α, with an amplitude bounded between −0.04 and +0.04, and the initial mutant population is 5.0 × 10^−6^ and 0 for seeds and adults, respectively. Populations of seeds and adults are, respectively, checked every 100 steps, and very small populations (<5.0 × 10^−8^) are supposedly extinct and removed from the simulation. Each set of simulations of the eco‐evolutionary dynamics is carried out on *R* 3.1.2 (R core team, 2013) and replicated for 20 times.

* Evolution of seed size*

Likewise, we rely on PIPs and numerical simulations to investigate the evolution of seed size. Procedures are identical to the scenario (i), except that seed dormancy α is fixed and mutations occur on seed size γ.

* Coevolution of seed dormancy and size*

PIPs cannot be applied to the context of coevolution, so we only rely on numerical simulations to understand the scenario. The simulation algorithm is similar with that of the scenarios (i) and (ii). The only difference was that in each time step, either phenotypical trait α or γ*,* can randomly mutate with an equal probability (i.e., half baseline mutation rate relative to the scenario (i) and (ii)).

### 2.3 Simulations of deterministic and stochastic environmental changes

Temperature, as a crucial environmental factor, was manipulated to evaluate the effects of environmental change on eco‐evolutionary dynamics. Three deterministic and two stochastic situations were simulated. Scenario I was set as the local patch temperature equal to the optimal germination temperature (*T*_*x*_ = *T*_opt_ = 25°C). We consider temperature shifts of 1.5°C (scenario II) or 3°C (scenario III) in local patches. As we use symmetric Gaussian functions, such shifts equivalently mimic warmer or colder situations relative to *T*_opt_.

In addition to these fixed and deterministic shifts on temperature, we also study scenarios in which random fluctuations occur. To simulate environmental uncertainties, we use white noise with mean optimal temperature of 25°C across years but variance within 1.5°C (scenario IV) or 3°C (scenario V).

## 3 Results

### 3.1 Ecological dynamics

**Figure 2:**
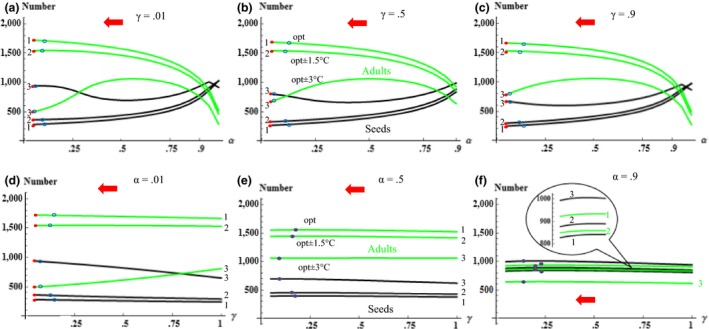
Ecological equilibria for a spectrum of seed dormancy (α, a–c) and of seed size (γ, d–f) when the alternative trait is fixed. Note: The red arrows indicate the evolutionary direction; filled red circles (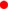) represent different evolutionary equilibria in respective conditions, while open blue circles (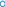) represent the values of evolved trait after simulations of 5.0 × 10^7^ steps (the fixed trait values are shown in each panel and the initial values of evolved trait (α or γ) are 0.5; seeds (black curves) and adults (green curves) are shown in pairs distinguished by alphabet numbers; that is, the same number means the number of seeds and adults in the same simulation condition

We first focus on the ecological dynamics without considering evolution. In Figure 2, we illustrate how the population structure changes with seed dormancy and size. Overall, given a seed size γ, levels of seed dormancy α have substantial influence on the state of ecological dynamics reflected by equilibrium densities (i.e., the number of seeds and adults) (Figure 2a–c). In general, given our parameter options, we get unbalanced populations and adults are more than seeds (Figure 2a–c). Symmetrically varying temperature around the opt (optimum) by 1.5 or 3°C, however, results in fewer adults accompanied by more seeds, and 3°C enables such a change in a higher amplitude than 1.5°C (Figure 2a, b, or c). The smaller the seed dormancy α, the larger the imbalance on the population structure (i.e., comparison of adult and seed densities) (Figure 2a, b, or c). Patterns are similar regardless of the seed size γ chosen, indicative of robustness of ecological dynamics in response to changes on α (Figure 2a, b, or c). By contrast, when seed dormancy α is fixed at 0.5, variation in seed size has minor influence on the population structure (Figure 2e). When temperature shifted by 1.5 or 3°C, the number of adults declines and that of seeds increases and the higher temperature deviation (i.e., 3°C) again results in the most balanced ecological structure (Figure 2e). This pattern is again consistent and robust, as it is observed at different levels of seed dormancy (compare Figure 2e with d or f).

### 3.2 Evolution of seed dormancy

**Figure 3:**
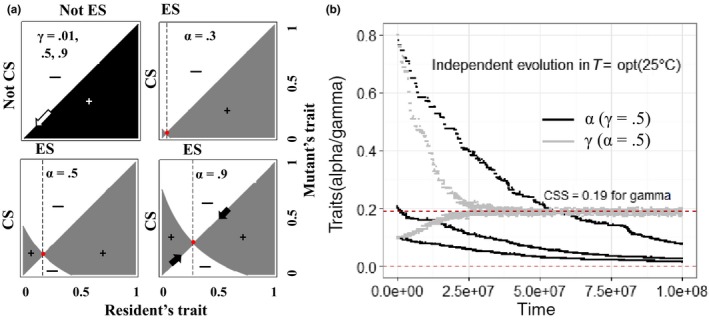
Pairwise invasibility plots (a) and evolutionary dynamics (b). Note: For A, the evolved morphs of seed dormancy and size are discriminated in black and gray, respectively; shades in black and gray depict leading eigenvalues (λ_*L*_) larger than one (marked by + sign; otherwise, marked by − sign), thus possibly invaded by mutants and its edges in cross shape represent λ_*L*_ equal to 1; ES and CS represent evolutionary stability and convergent stability, respectively; For B, one convergent and noninvasible singularity exists for seed size, marked in red dashed line and termed continuously stable strategy (CSS, which corresponds to an evolutionarily stable equilibrium where no evolutionary dynamics exist); no ESS (i.e., noninvasible singular strategies) exists for seed dormancy and it evolves toward zero, marked in red dashed line

The pairwise invasibility plots (PIPs) show that seed dormancy is always counter‐selected under the assumption of our model (Figure 3a, black PIP). PIPs are corroborated by simulations, also showing that seed dormancy α is selected against (Figure 3b, black curves). As expected, evolutionary dynamics are progressively faster when starting at high dormancy (α = 0.8) than at low dormancy (α = 0.2 and 0.1) (Figure 3b).

**Figure 4:**
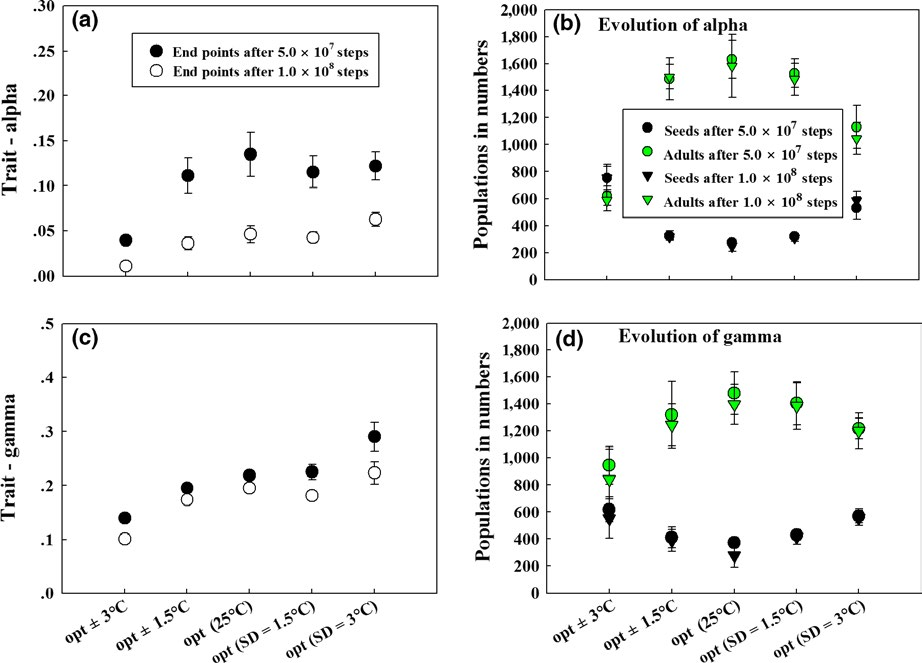
Evolutionary end points and corresponding number of populations for the independent evolution of seed dormancy (a,b) and size (c,d) after numerical simulations of 5.0 × 10^7^ and 1.0 × 10^8^ steps. Note: The error bar for each end points or the number of populations (i.e., seeds or adults) is calculated by 20 replicates for each set of simulations; sd represents standard deviation (i.e., temperature variation); the values of fixed trait and the initial evolved trait (α or γ) are 0.5; graphic representation of the simulation dynamics was provided in Figs. S1 and S2

Given the observed results in Figure 2a–c, where we now report the direction of evolutionary changes (red arrows), evolution, via pushing seed dormancy toward smaller values, generally decreases the balance between adult and seed densities. While seed dormancy α is always counter‐selected, the speed of dormancy evolution for different scenarios is compared by showing evolutionary states after 5.0 × 10^7^ and 1.0 × 10^8^ steps (empty and filled circles, respectively), given the probability of dormancy starting at 0.5 (Figure 2b). We note that evolutionary speed changes depending on temperature shifts with important consequences for the ecological structure (Figure 2b). At the opt where species are locally adapted, seed dormancy evolves more slowly than in maladapted environments (opt ± 1.5 or 3°C), indicating that the speed of evolution is the fastest in nonopt environments where seed dormancy is more counter‐selected (Figures 2b and 4a). Such a relationship remains when simulation steps are extended to 1.0 × 10^8^ times (Figures 2b, 4a and S1). While the evolution of dormancy generally makes the population structure more unbalanced, at opt ± 3°C where species are largely not adapted, evolution first increases then decreases the amount of adults while eventually increases the amount of seeds, resulting in a balanced distribution of the two stages and decreased total populations (Figures 2b and 4b). These results are observed, regardless of the values at which γ is fixed (Figure 2a,c).

Due to these differences in evolutionary speed, for a given simulation time, evolved dormancy α is lower in opt ± 1.5°C than in opt and lowest in opt ± 3°C and there are no qualitative changes on such a pattern when sets of parameter values are randomly tweaked up‐ or downward (Fig. S3I‐A). This indicates that shifts in the environment suppress seed dormancy to lower values in the case of our model.

### 3.3 Evolution of seed size

Pairwise invasibility plots show that, in consideration of the evolution of seed size γ, only one singularity exists in each case. This singularity is convergent (i.e., given a resident strategy, mutant strategies closer to the singularity are favored) and not invisible (i.e., when the singularity is reached, no mutant can invade), thus a continuously stable strategy (CSS) (Christiansen, 1991; Eshel, 1983) (Figure 3a, gray PIPs). As a result, the evolution of seed size eventually leads to this point (Figure 3b, gray curves). High seed dormancy increases the selected seed size value (compare the three gray PIPs in Figure 3a). The observed evolutionary dynamics are consistent with the analysis of PIPs (Figure 3b, gray curves).

Based on the pattern analyzed in Figure 2d–f on evolutionary directions and equilibria, the evolution of seed size only has minor effects on the structure of the population at equilibrium. Given low dormancy α (<0.3), small seed sizes γ are inclined to be counter‐selected (Figures 2d and 3a). Analogously, the speed of seed size evolution for different scenarios is compared using its evolutionary endpoints after numerical simulations for 5.0 × 10^7^ and 1.0 × 10^8^ steps. Evolution of γ is close to CSS points before 5.0 × 10^7^ steps (Figure 2e). Evolved seed size is higher for well‐adapted phenotypes, compared with phenotypes experiencing temperature shifts of 1.5 or 3°C (Figure 2e). Evolution of seed size γ ends in fixation at CSS points for more repeated simulations of 1.0 × 10^8^ steps (Figures 4c and S2). Temperature shifts have distinct influence on ecological structures in the evolution of seed size. More precisely, as temperature shifted by 1.5 or 3°C, the number of seeds increases while that of adults decreases but adults always outnumber seeds (due to low seed persistence assumed) and the total population declines (Figures 2e and 4d). We finally note that the foregone results are robust, given different values of seed dormancy α (Figure 2d and f, also see Fig. S3I‐B for other robustness tests).

### 3.4 Coevolution of seed dormancy and size

**Figure 5:**
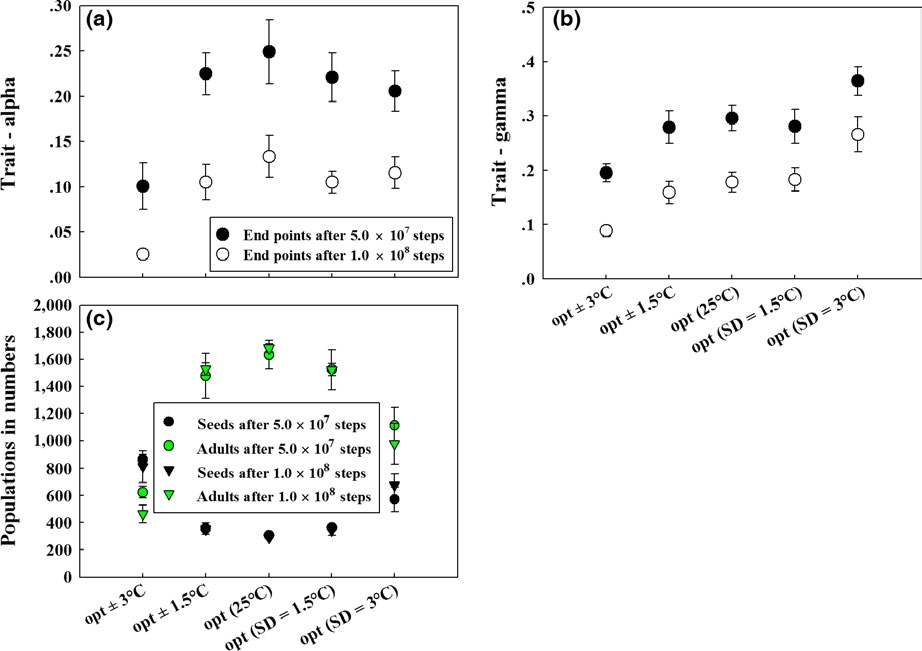
Evolutionary end points and corresponding number of populations for the joint evolution of seed dormancy and size after a numerical simulation of 5.0 × 10^7^ and 1.0 × 10^8^ steps. Note: The error bar for each end points or the number of populations (i.e., seeds or adults) is calculated by 20 replicates for each set of simulations; sd (in axis labels) represents standard deviation (i.e., temperature variation); the initial matrices (α, γ) and (seeds, adults) are (0.5, 0.5) and (5, 5), respectively; graphic representation of the simulation dynamics was provided in Fig. S4

Should seed dormancy and size jointly evolve, selected seed size γ and dormancy α gradually decline (Figure 5). This may be easily explained by the two previous scenarios on “independent” evolution; as seed dormancy is always counter‐selected and evolved seed size becomes lower when the value of seed dormancy decreases (Figure 3), coevolution simply leads to ever‐decreasing values for the two traits (Figures 5a,b and S4A). Compared with the independent evolution, seed dormancy and size evolve almost at the same speed (compare empty circles in Figure 5a,b and filled circles in Figure 4a,c keeping in mind that the effective mutation rate is half in coevolution relative to independent evolution). Corresponding ecological dynamics (Figures 5c and S4B) are consistent with trends observed given the ecological equilibrium status (Figure 2). Specifically, adults outnumber seeds in opt, temperature shift by 1.5°C, while seeds exceed adults in temperature shift by 3°C, which resembles the ecological structure in the evolution of seed dormancy only (Figures 5c and 2a,b). This indicates that the evolution of dormancy has substantial influence on the evolution of the two traits and ecological systems.

### 3.5 Effects of stochasticity

As the simulation time extended (i.e., 1.0 × 10^8^ steps), increased temperature variation (i.e., within 3°C) ends up in higher end points of α or γ relative to their counterparts in nonvarying (opt) scenarios (Figure 4a,c). This indicates that the evolved trait (α or γ) has a palpable effect on bet‐hedging (i.e., reduced speed in counter selection) especially for the elevated amplitude of temperature fluctuations and in the long term. Furthermore, temperature variation alters the ecological structure, where the relative frequency of adults and seeds becomes more balanced with higher amplitude of temperature variation (Figures 4b,d and S1, 2).

In the scenarios of joint evolution, selected seed sizes are always higher in increased temperature variation (i.e., within 3°C) than in opt (Figure 5b). However, evolved seed dormancy phenotypes are on average lower in temperature variation than in opt (Figure 5a). This suggests that seed size evolves more slowly than dormancy under environmental uncertainties when compared with the scenario in opt. Moreover, coevolution alters the ecological system such that adults and seeds become more balanced with higher degree of temperature variation (Figures 5c and S4B).

## 4 Discussion

While pieces of previous work deal with the evolution of either seed dormancy or size (e.g., Cohen (1966); Venable and Brown (1988)), we here investigate existing feedbacks between the two traits as well as their joint evolution. Also, we explicitly tackle effects of climate change in this evolutionary context rather than solely focusing on fixed environmental settings. Besides evolved traits, we also elucidate how population structures alter over time given different evolutionary strategies for species that are thermally adapted or nonadapted. Analyses of evolutionary speed for the evolved traits allow evolutionary rescue of populations in face of climate change. This study is therefore able to advance our understanding of eco‐evolutionary feedbacks that shape and maintain biodiversity in the context of climate changes.

### 4.1 Temperature shifts and life‐history evolution

Regardless of whether seed dormancy α and size γ jointly or independently evolve, selection gives rise to faster evolution when species are not locally adapted (i.e., temperature shifts by 1.5 or 3°C) (Figures 4a,c and 5a,b). Climate change could directly select for higher levels of seed dormancy through increasing the probability of bad years. However, our results do not follow this expectation, as temperature shifts in fact select for lower levels of dormancy (Figures 4c and 5c). A possible explanation relies on variations in density‐dependent effects. In our model, seed germination is directly affected by the competition among seedlings and while temperature shifts indeed worsen the environment (thereby selecting for more dormancy), it also globally decreases the number of seeds, thus relaxing the competition at the germination stage. This indirect effect creates positive effects for more germination and thus less dormancy.

These implications that global change directly and indirectly affects the selection of dormancy levels are in line with empirical and experimental evidence. Soil temperature has been shown to largely impact the synchronization of seed germination in the soil seed bank (reviewed in (Finch‐Savage & Leubner‐Metzger, 2006)). Consistent with our results, increased temperature or decreased elevation that is ascribed to elevated temperature as well as decreased precipitation and soil moisture promotes dormancy loss (Ooi, Auld, & Denham, 2012; Zhou & Bao, 2014). Note, however, that germination processes do not simply depend on temperature effects. For example, moist‐chilling is a common dormancy‐breaking stimulus for imbibed mature dormant seeds in natural stands, while under some conditions, extended chilling can result in secondary dormancy (i.e., nondormant seeds fail to germinate due to reentering dormant state by unfavorable cues for germination) (Penfield & Springthorpe, 2012). In such conditions, temperature shifts may increase the time to germination due to insufficient dormancy decay or re‐induction to dormancy. This mechanism is important in the plant life cycle and can be easily included in future versions of the model, for instance by modifying the environmental constraints in the *G*_*j*_ equation (see the Model section), to incorporate moisture effects in addition to temperature effects, as well as the possibility of secondary dormancy.

Temperature and other selective pressures pertaining to temperature also affect seed size evolution (Vidigal et al., 2016). Our results imply an overall decrease in seed sizes with temperature shifts. In concordance with this, increasing temperature during growth has been shown to reduce nutrient and water availability, which in turn lower seed size (Wulff, 1986). Low elevation with higher temperature has also been suggested to lead to smaller seeds (Vidigal et al., 2016; Zhou & Bao, 2014). More generally speaking, small seeds are superior colonizers and large seeds are superior competitors.

Equally important is the stochasticity of temperature to the evolution of seed dormancy and size. With wide temperature variation (e.g., 3°C relative to 1.5°C) between generations, species undergo high variance in the fitness and thus bet‐hedging effects give rise to low germination fractions and/or large seed size (better provisioning to survive harsh settings) (Figures 4a,c and 5b). This result is evidenced by previous theoretical investigations (Ellner, 1985a, 1985b; Gremer & Venable, 2014).

### 4.2 Impact of joint and independent evolution

If seed dormancy α and size γ jointly evolve, both are counter‐selected in our model (Figures 5a,b and S4A). In their independent evolution, seed dormancy and size evolve as fast as in coevolution (Figures 5a,b and 4a,c); seed size gets to a selective strategy while dormancy is selected against (Figures 3 and 4a,c). These indicate that in the coevolution scenarios, the evolution of seed size dampens but does not alter the counter selection of seed dormancy, and eventually, seed dormancy and size are selected against.

Long‐lived species buffered from temporal variation in the environment often exhibit less dormancy (Rees, 1994; Venable & Brown, 1988). Nonetheless, seed dormancy is not an all‐or‐nothing trait. Contrary to what is observed in our result, environmental uncertainty (Bulmer, 1984; Cohen, 1966) and/or competition (such as density dependence) in fluctuating environments (Ellner, 1987) have been shown to favor seed dormancy. The potential agent of selection, high precipitation or a low amount with substantial fluctuation between generations, selects for dormancy (Volis & Bohrer, 2013). Also, a negative correlation between seed dormancy and size is generally observed (Grime et al., 1981; Kiviniemi, 2001; Larios et al., 2014; Rees, 1996; Thompson & Grime, 1979; Vidigal et al., 2016), such that when dormancy evolves to a small value, seed size should evolve toward a large value. We do not observe selection of increased dormancy, nor do we get a negative correlation between seed size and seed dormancy. This inconsistency between our results and empirical observations may rest on the fact that in our model, populations reach stable equilibrium densities (compared with (Gremer & Venable, 2014; Gremer, Kimball, & Venable, 2016)), such that deep dormancy cannot be selected as a bet‐hedging strategy to reduce mortality due to density‐dependent mortality or to direct variations in the environment. In fact, when no selective forces impose on dormancy, dormancy turns into a supplementary source of mortality. Moreover, the counter selection of seed dormancy due to extra costs is imposed on seed survival in our model. Deep seed dormancy may be selected for given high seed survival rate in the soil seed bank (Cohen, 1966; Gremer & Venable, 2014; Venable & Brown, 1988) and/or a decreased density‐dependent effect (Ellner, 1985a, 1985b; Gremer & Venable, 2014). Additionally, we found that when secondary dormancy is incorporated into the model (i.e., a portion of nondormant and nongerminable seeds becomes dormant and goes into the soil seed bank), the model may select for certain levels of dormancy (results of another modified model not detailed here). These results indicate that low seed persistence invariably selects against seed dormancy, and increasing seed persistence may alter dormancy evolution in stable systems and thus the correlation between seed dormancy and size.

In our model, considering long‐lived species exposed to density‐dependent effects, we see that joint evolution results in low dormancy and small seed size. When dormancy decreases, germination increases at the cost of seed persistence and the adult population does not significantly change if the seed population can be maintained. In our model, smaller seeds allow higher fecundity (Fig. S5A) for a given total reproductive investment (Fig. S5B). Increasing fecundity through smaller seeds sustains the seed population while decreasing seed persistence. This represents one evolutionary scenario leading toward quick germination and smaller seed size at the cost of seed persistence. In nature, the soil seed bank is more associated with annuals than perennials, which is supported by comparative studies (Rees, 1993, 1996; Thompson, Bakker, Bekker, & Hodgson, 1998). Dormancy can evolve differently in perennials, as there are other sources of variabilities, such as fire (Liu et al., 2016). This also suggests that evolutionary forces do not necessarily favor large seed size to increase seed persistence in the soil seed bank. It is worth noting that, if large seeds are selected for, seed dormancy is not likely to be always selected against, in the sense that seed persistence has high benefits.

In perennial plants, the combination of traits (high fecundity, small seeds, low seed persistence, and low dormancy) we observe corresponds to some species in nature (e.g., aspen (*Populus tremula* L.) and fireweed (*Chamerions angustifolium* (L.) Holub)). As such, our model leads to strategies akin to the life history of some opportunistic species, which are more effective in exploiting ephemeral ecological opportunities. Aspen, for example, has high seed production capacity (1,000–1,500 seeds/catkin and as many as 40,000 catkins/tree). Seeds of aspen are very small and light (~0.06 to 0.17 g/thousand‐grains), which helps dispersal over long distances, and its germinability after maturation is usually fast and high (70%–95%), but its viability decreases after dispersal and this corresponds to the transient seed banks of aspen (Thompson, Bakker, & Bekker, 1997).

### 4.3 Population structures

In our model, even when species are well adapted, adults exceed seeds in numbers (Figures 2, 4 and 5) due to high and increased seed mortality as evolution proceeds. Temperature shift robustly leads to more balanced population structures (Figure 2), while evolution increases the imbalance in population structures particularly in opt or slight temperature shift (i.e., 1.5°C) (Figure 2). Considering the interaction of evolution and temperature shifts or variation, the population structure becomes more balanced, while the total population decreases (Figures 4b,d and 5c).

Temperature shifts impose extra costs on germination and these determine whether the seed‐adult system can be sustained in the unbalanced structure (i.e., compared with the constant adaptive scenario). Apparently, temperature shifts causing maladaptation do not increase population density (i.e., total number of seeds and adults). As the population structure becomes more balanced in temperature shifts, there must exist critical points (i.e., combinations of temperature and evolved trait(s)) at which seed and adult density are equal, evidenced by contrasting ecological dynamics when temperature shifts by 1.5 or 3°C (Figures 2, 4b,d, and 5c). In interaction with evolved traits, high temperature shifts (e.g., 3°C) largely affect population structure, which is facilitated when the evolution of seed dormancy is allowed (Figs. S1, S2, and S4B). The evolution of dormancy has indeed a large influence on the population structure and in conjunction with temperature shifts, which results in seed numbers superior to adults. This process is attained mainly through altering fecundity and germination. The probability of germination greatly decreases at high temperature shifts, resulting in fewer adults (note that constant adult survival is assumed), while elevated fecundity due to decreased adult density‐dependent competition (and predictably smaller seeds, if seed size evolves or coevolves with seed dormancy) resulting in more seeds (note that seed survival deteriorates due to counter‐selected seed dormancy).

Our results illustrate that climate change not only has direct impacts on population structures (as already observed (Walther et al., 2002; Clark et al., 2014)), but also shows how evolutionary trajectories may exacerbate these changes. Moreover, the soil seed bank can help balance population dynamics by spreading risk and allowing population recovery after disturbance (Grime, 1989), while global warming leads to decreased seed persistence (Childs et al., 2010; Ooi et al., 2009). In response to temperature shifts or variations of high magnitude, significant changes on ecological structures (e.g., from an unbalanced to a more balanced state) occur in stable systems, indicating that life history changes significantly and may gradually lack the power of resilience (no trace of resistance and recovery in simulation results), thus becoming more vulnerable to collapse.

### 4.4 Perspectives

Seed dormancy is an intrinsic attribute affecting regeneration dynamics and seed size acts as one of the vital determinants for the evolution of seed dormancy. While the goal of the present model is to better understand their covariation in isolation, an important perspective is to account for explicit spatial aspects. These spatial aspects are especially important in the global change context, as temperature shifts depend on latitude and altitude gradients and species dispersal to higher altitudes and latitudes is thought to be a major constraint to their future survival. Also, spatial context influences gene flows and evolutionary dynamics with again important consequences for species competition and survival (Norberg, Urban, Vellend, Klausmeier, & Loeuille, 2012). The two traits we studied here are intrinsically related to seed dispersal such that a spatially explicit context should modify our results. While this study uncovered seed dormancy as a means to disperse in time, seed dispersal is another important means to dispersal in space and also a risk‐spreading strategy (Buoro & Carlson, 2014; Cohen & Levin, 1987). They may evolve as phenotypic plasticity (e.g., bet‐hedging) (Gomez‐Mestre & Jovani, 2013; Philippi & Seger, 1989; Slatkin, 1974) to reduce parent–offspring conflict, kin competition, and local extinction (Ellner, 1986; Gremer & Venable, 2014; Vitalis, Rousset, Kobayashi, Olivieri, & Gandon, 2013), thus promoting adaptation, stability, and persistence (Kovach‐Orr & Fussmann, 2013). Consequently, selection acts on trade‐offs in temporal and spatial dispersal and eventually maximizes fitness (Buoro & Carlson, 2014). This study is therefore a springboard toward more integrative scenarios aiming to better forecast the evolution of life‐history traits in temporally and spatially variable environments.

## Conflict of Interest

None declared.

## Data accessibility

This study does not use data.
