# Supplementary material for: Impact of temperature shifts on the joint evolution of seed dormancy and size
Source: Ecol Evol. 2016 Nov 27;7(1):26–37. doi: 10.1002/ece3.2611 (PMC5216621; doi:10.1002/ece3.2611)
Supplement: Supplementary file 1 [file ECE3-7-26-s001.docx]

Supplementary material

Impact of temperature shifts on the joint evolution of seed dormancy and size

Yang Liu^1,*^, Sébastien Barot^2^, Yousry A. El-Kassaby^1^, Nicolas Loeuille^2^

^1^Department of Forest and Conservation Sciences, University of British Columbia

2424 Main Mall, Vancouver, British Columbia V6T 1Z4 Canada

*^2^Sorbonne Universités, UPMC Université Paris 06, CNRS, IRD, INRA, Université Paris Diderot, Institute of Ecology and Environmental Sciences (UMR7618), 7 quai St Bernard, 75005 Paris, France*

**Running title:** Seed evolution under global change

^*^Author for correspondence:

Yang Liu

Email: [yliu2011@interchange.ubc.ca](mailto:yliu2011@interchange.ubc.ca)

**Figure S1** Graphic representation of eco-evolutionary dynamics of seed dormancy (evolved trait and populations) in a span of simulations of 1.0×10^8^ steps

Note: different curve colors for trait or different dot colors for populations represent simulation replicates in a total of 20; the values of fixed trait (γ) and the initial evolved trait (α) are 0.5; in the course of mutant emergence and demise, the values of evolved traits and population (seed and adult) sizes were recorded every 100 steps of each simulation.

**Figure S2** Graphic representation of eco-evolutionary dynamics of seed size (evolved trait and populations) in a span of simulations of 1.0×10^8^ steps

Note: different curve colors for trait or different dot colors for populations represent simulation replicates in a total of 20; the values of fixed trait (α) and the initial evolved trait (γ) are 0.5; in the course of mutant emergence and demise, the values of evolved traits and population (seed and adult) sizes were recorded every 100 steps of each simulation.

**Figure S3** Eco-evolutionary dynamics of each trait (seed dormancy [I-A] and size [I-B]) and their graphic representation (II) in opt or temperature shifts and robustness analysis

Note: simulations were run using different parameters values and the initial α and γ were 0.5 for both; the following parameter values (seed and adult survival) were replaced for corresponding ones in the main text: 1, *B*=4/5; 2, *p*=0.85; 3, *V_A0_*=0.94; 4, *p*=0.75; 5, *V_A0_*=0.9; 6, *B*=2/5, and three different values of α or γ were also implemented in the model (the one used in this study was highlighted in red); due to no ESS for seed dormancy, the end point of α was used for comparison after simulations of 5.0×10^7^ steps and the evolution of γ almost can get to its CSS, if it exists, after 5.0×10^7^ steps; graphic representation of the simulation dynamics for the evolution of α was provided in Figure S3II; the replacements are provided in the top-right corner of each panel; the red dashed line (top one) denotes the evolutionary end point of α for T=opt (25°C) using the parameter values in the main text, while another red dashed line (bottom) denotes the evolutionary final point of α (i.e. 0) for T=opt (25°C).

**Figure S4** Graphic representation of eco-evolutionary dynamics of joint evolution of seed dormancy and size (evolved traits (A) and populations (B)) in a span of simulations of 1.0×10^8^ steps

Note: different curve colors for trait or different dot colors for populations represent simulation replicates in a total of 20; the values of the initial evolved traits (α or γ) are 0.5; in the course of mutant emergence and demise, the values of evolved traits and population (seed and adult) sizes were recorded every 100 steps of each simulation.

**Figure S5** Fecundity without considering density-dependent competition as a function of seed size (A) and trade-off of plant total reproductive investment and seed dispersal-related survival (B)

Note: red arrows indicate the evolutionary direction.

**Figure S1**

**
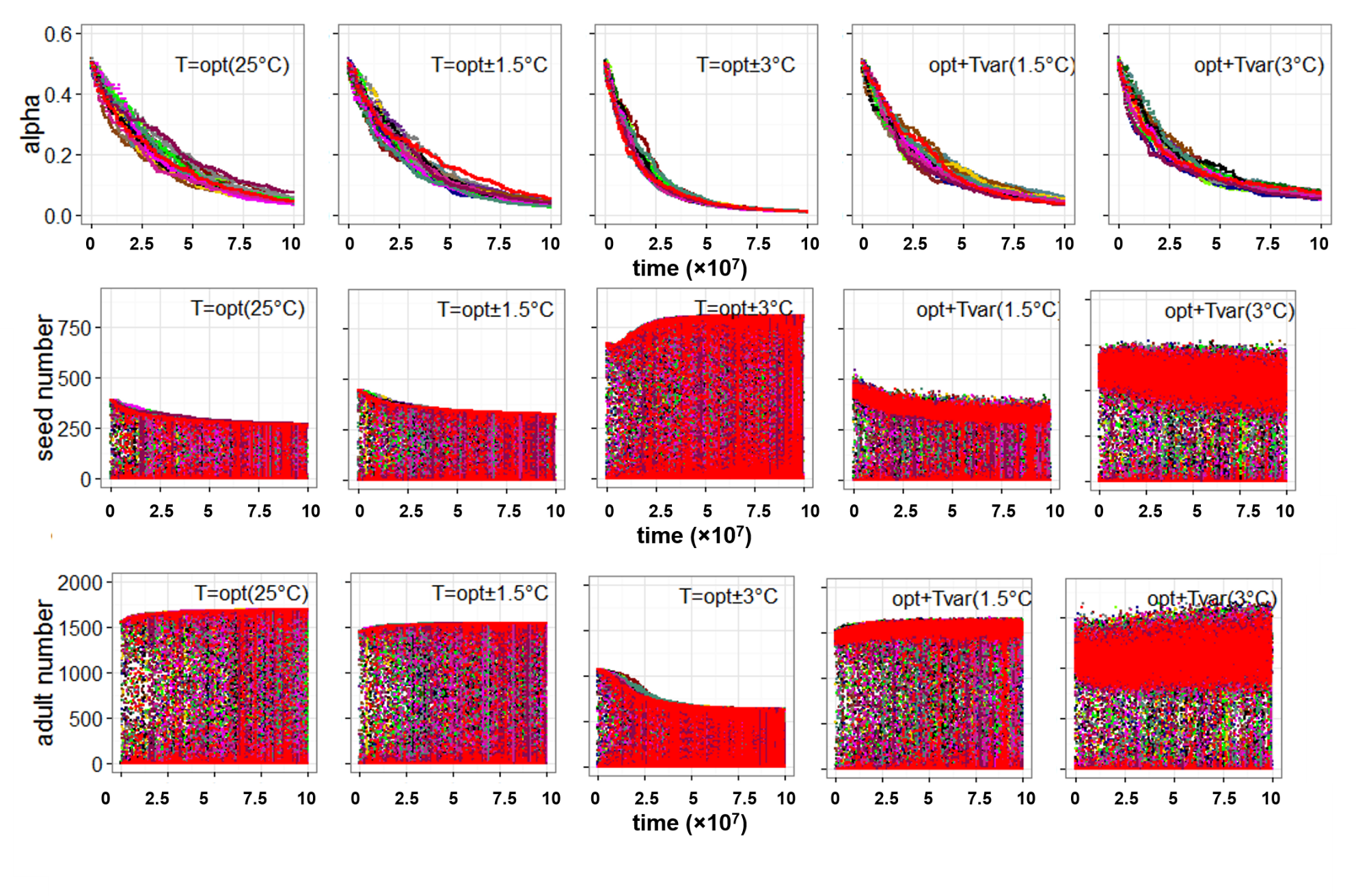
**

**Figure S2**

**
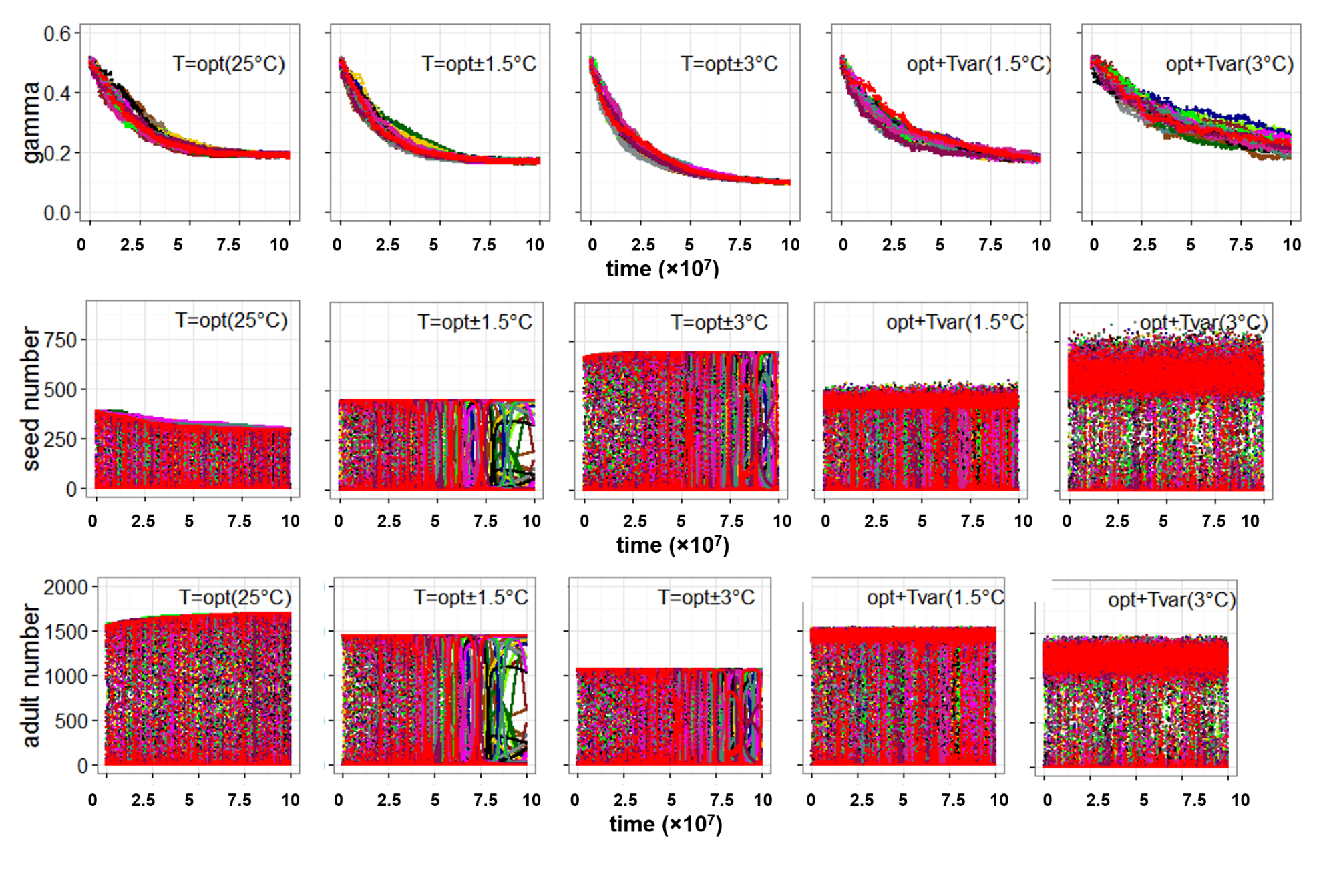
**

**Figure S3**

**I)**

**
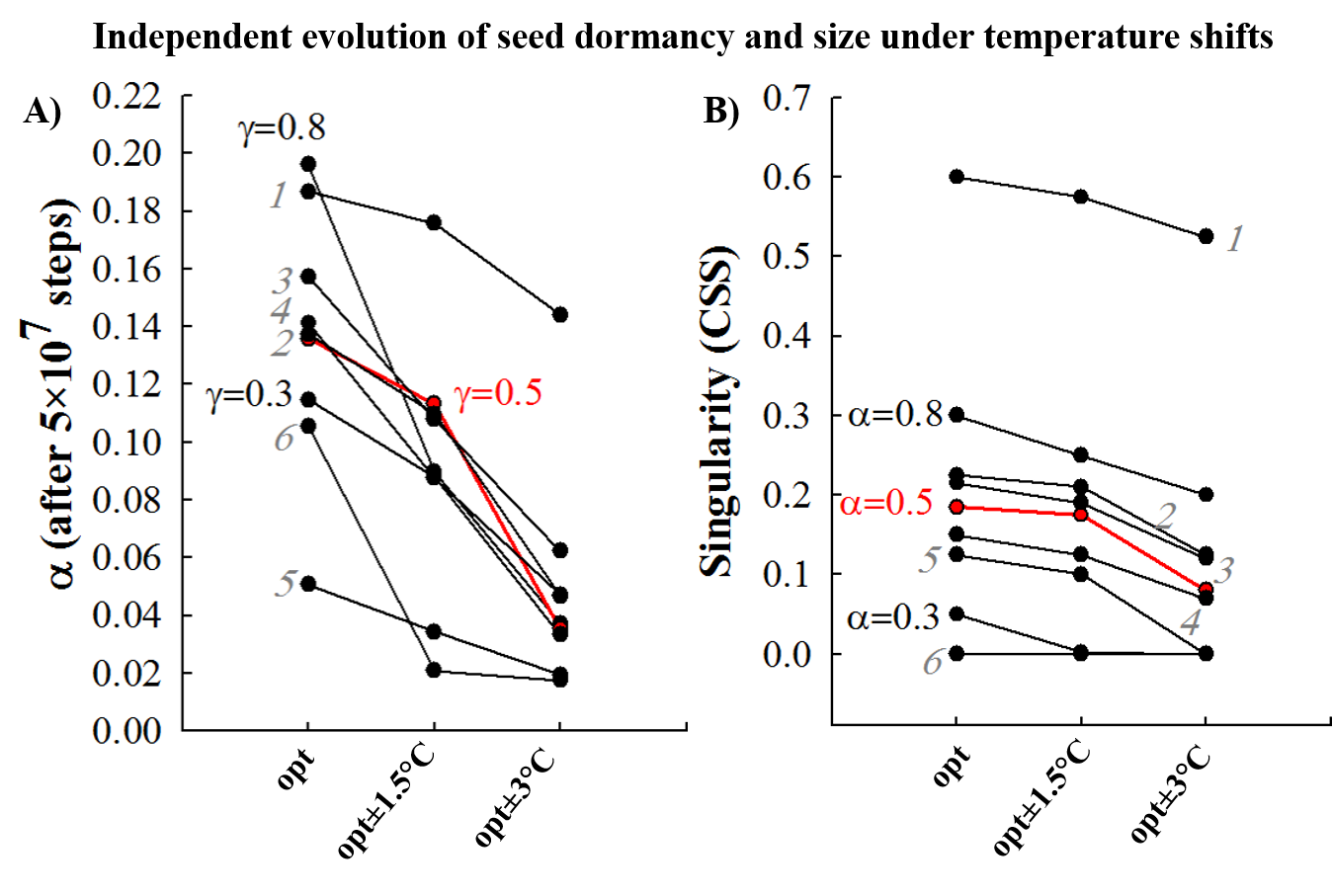
**

**II)**

**
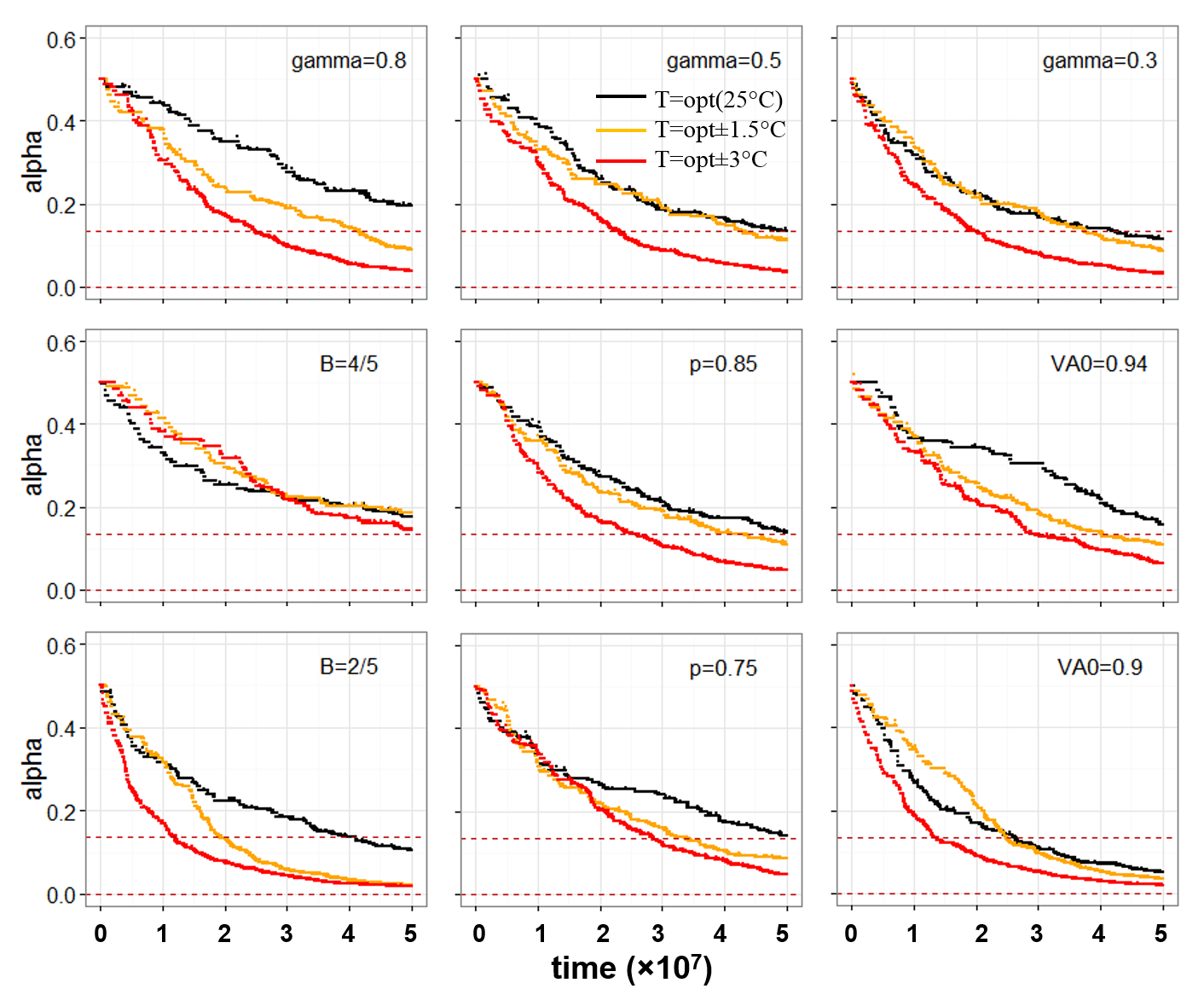
**

**Figure S4**

**A)
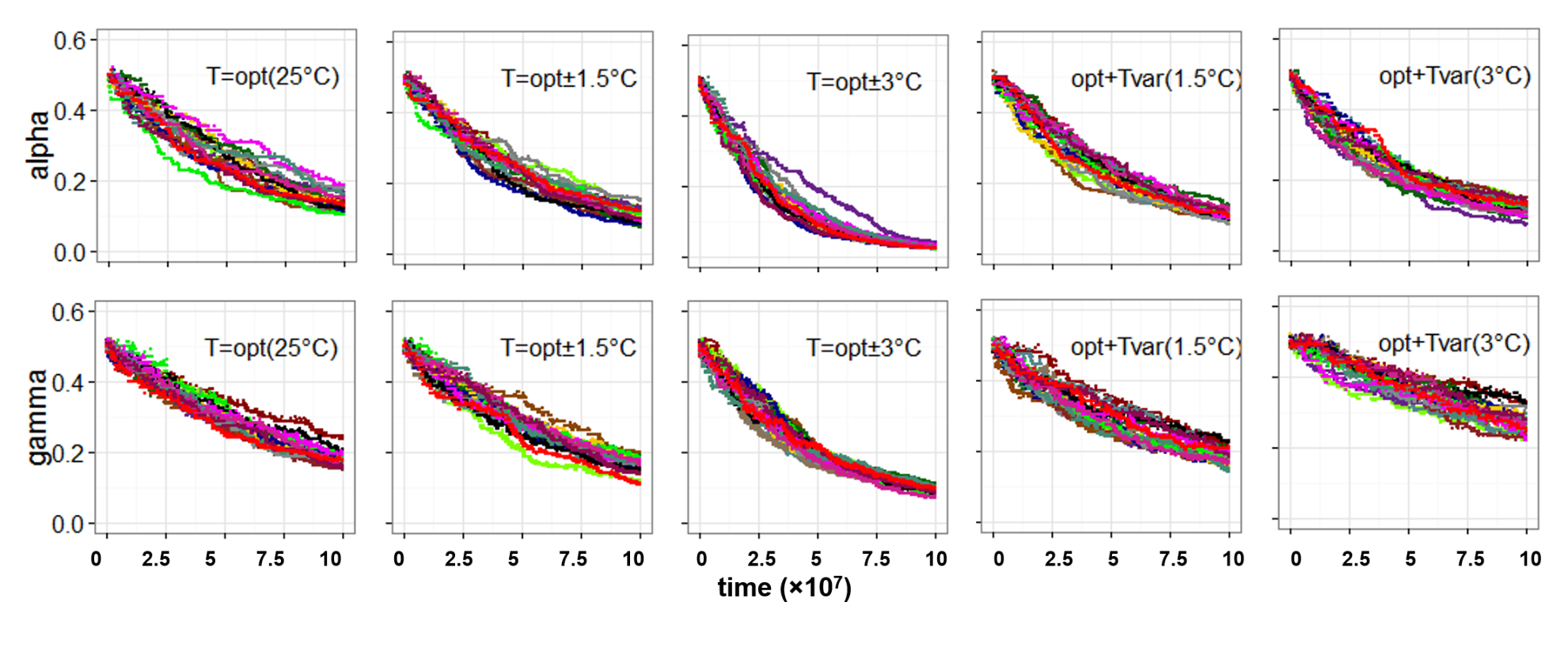
**

**B)**

**
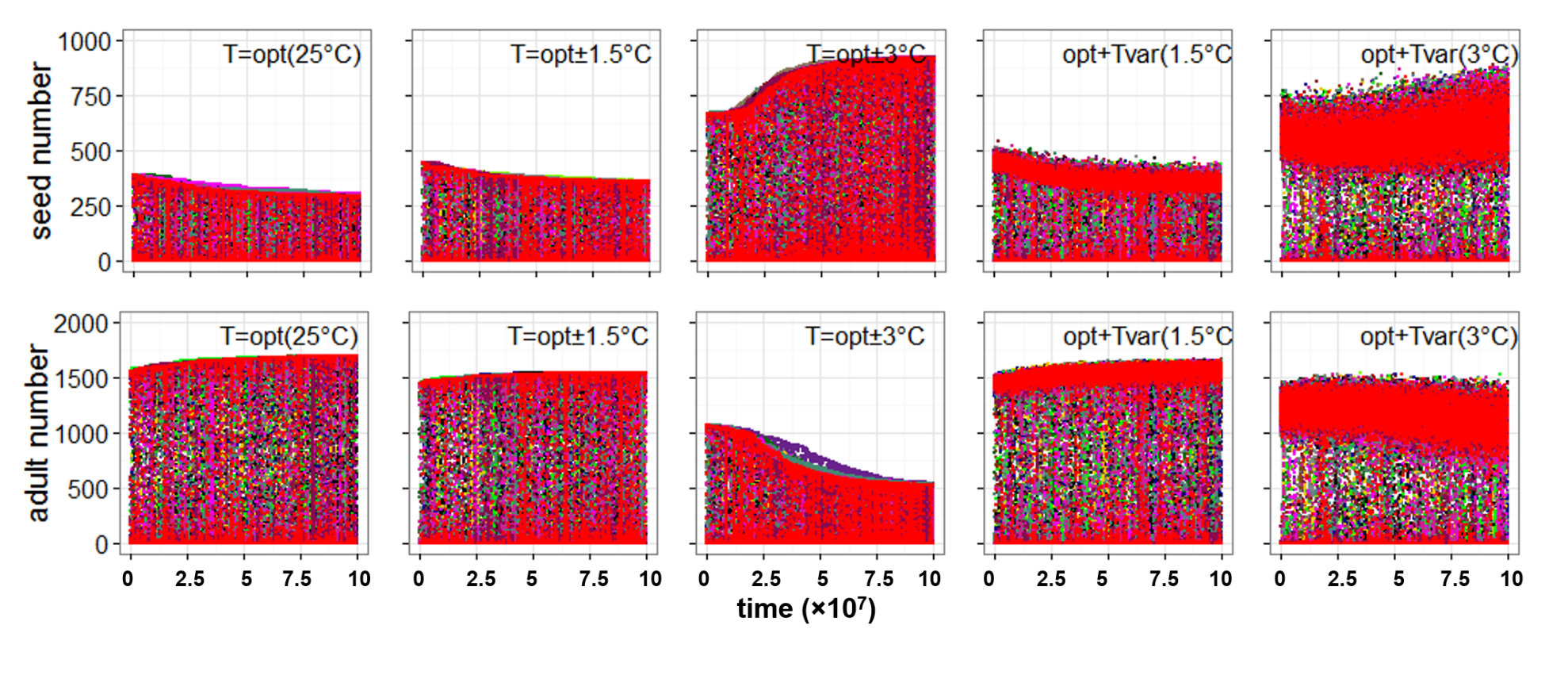
**

**Figure S5**

**
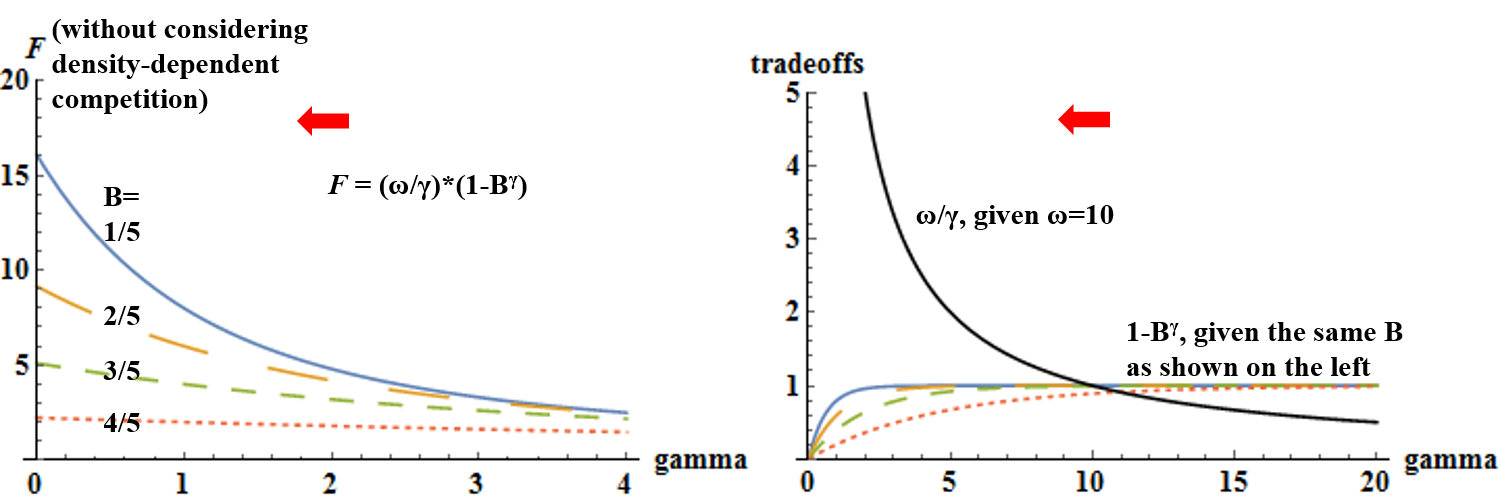
**

**B**

**A**
